# Primary Cutaneous Leiomyosarcoma of the Lower Extremity: A Case Report and Literature Review

**DOI:** 10.7759/cureus.14282

**Published:** 2021-04-03

**Authors:** Victor Chalfant, Tyler Schriber, Ahmed Sabri, John Gossen, Darren Groh

**Affiliations:** 1 Department of Pathology, Creighton University School of Medicine, Omaha, USA

**Keywords:** neoplasm recurrence, sarcoma soft tissue, cutaneous leiomyosarcoma, mohs surgery, wide local excision, h&e staining, desmin, vimentin, smooth-muscle actin, dermatopathology

## Abstract

Cutaneous leiomyosarcoma is a rare soft-tissue sarcoma that appears non-specific clinically and often is misdiagnosed as squamous cell carcinoma. We report the case of a 59-year-old Caucasian male with a grade I leiomyosarcoma tumor on his lower extremity with no previous history of local trauma. The tumor is composed of highly atypical spindle cells with pleomorphic nuclei and mitotic activity on hematoxylin and eosin stains. The diagnosis is confirmed with immunohistochemistry staining positive for smooth muscle actin, vimentin, and desmin. Due to high recurrence rates, the prognosis for leiomyosarcomas remains poor and requires close follow-up to prevent progression.

## Introduction

Cutaneous leiomyosarcoma is a rare neoplasm, with an annual incidence of 0.2 per 100,000, commonly occurring in the sixth and seventh decades of life. These tumors are most commonly found on the extremities, and also are found often on the trunk, or head and neck, presenting as asymptomatic nodules that may or may not have erythema or tenderness [1]. The two types of leiomyosarcomas are leiomyosarcomas arising from the arrector pili muscles and subcutaneous leiomyosarcomas arising from the adipose tissue’s vascular smooth muscle. Because of this, subcutaneous leiomyosarcomas are usually larger, staged higher, and subsequently more aggressive. For either subtype, the prognosis is worse when the larger tumor is diagnosed [2]. A biopsy is necessary for the proper diagnosis of cutaneous leiomyosarcomas, as well as distinguishing the true depth of the lesion. Immunohistochemistry will show vimentin and smooth muscle actin in all cases while showing muscle actin and desmin in over half of cases [3]. After diagnosis, treatment is most often wide local excision or rarely Mohs micrographic surgery. The use of adjuvant chemotherapy and immunotherapy is still controversial, and an area of continued study, but is the normal course when resection is not possible [1,4]. There has been shown to be a sizable recurrence rate, greater than 24%, for both subtypes, though the malignancy rate for the subcutaneous subtype is much higher, which tends to need closer follow-up [5]. Here we present the rare case of a cutaneous leiomyosarcoma presenting symptomatically, without metastasis, with a focus on efficient diagnosis and surgical treatment.

## Case presentation

**Figure 1:**
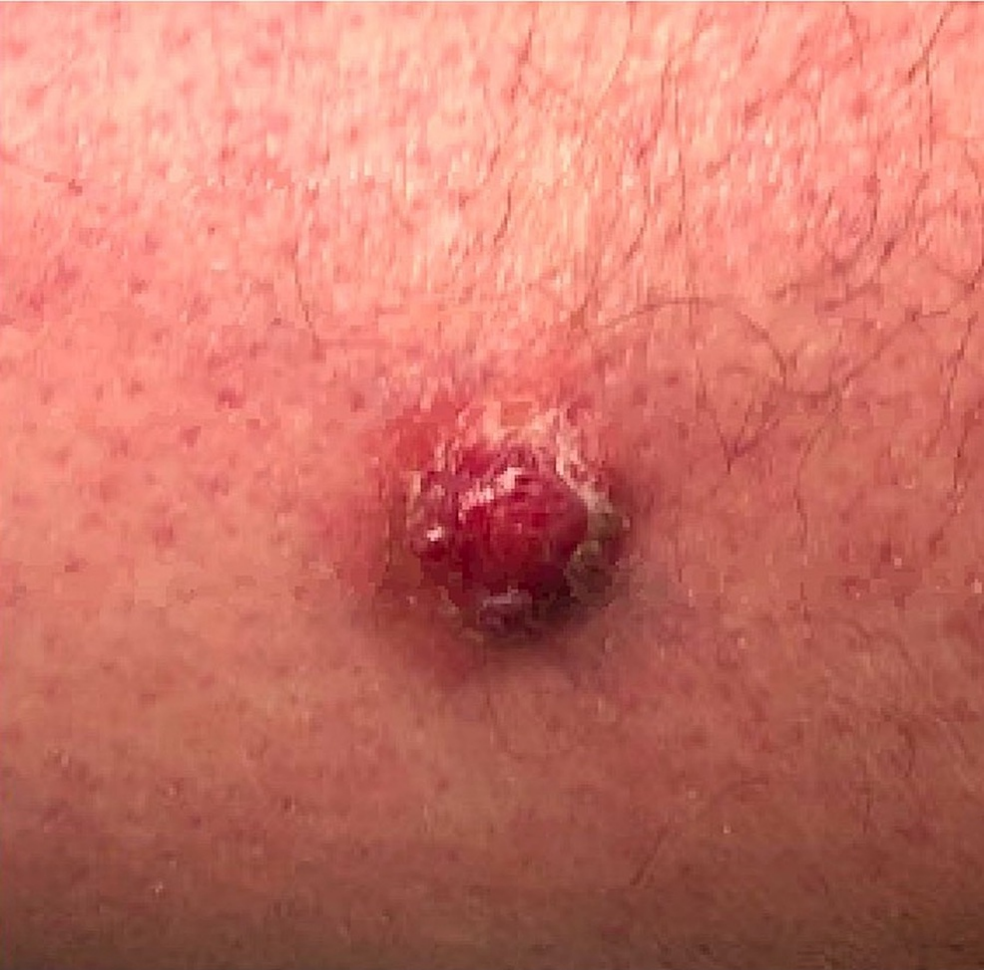
1.4 cm purplish eroded lesion on the right anterior thigh.

A 59-year-old Caucasian man with a past medical history of chronic obstructive pulmonary disease is referred by his primary care physician for a suspicious skin lesion measuring 1.4 cm on his right anterolateral thigh due to concern of it increasing in size over the past year and starting to become significantly tender (Figure 1). The patient noticed the lesion over a year ago. The patient reports the use of 325 mg tablets of acetaminophen every six hours as needed for pain. The patient denies taking any medication regularly. The patient denies any associated fatigue, unexplained weight loss, bone pain, fever, nausea/vomiting, or changes in his bowel/bladder habits. The patient reports no past history of intense sun exposure or history of skin cancer. In social history, the patient denies usage of alcohol, however, reports an 88-pack-year of smoking. The patient has no family history of cancer in first-degree relatives. The area around the lesion is anesthetized with 1 ml of 2% xylocaine with epinephrine. A subsequent 8 mm shave biopsy of the lesion is taken by an electrodessication and curettage (ED&C) technique. The biopsy is placed in buffered formalin and sent for biopsy. Antibiotic ointment is applied and a sterile dressing placed.

**Figure 2:**
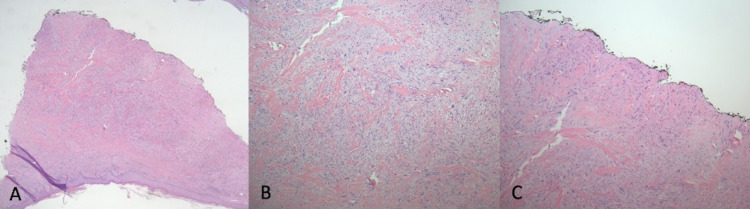
Hematoxylin and eosin (H&E) stains under low power view at 4x (A), 10x (B), and of the biopsy site showing the tumor extending into the deep margin at 10x (C).

**Figure 3:**
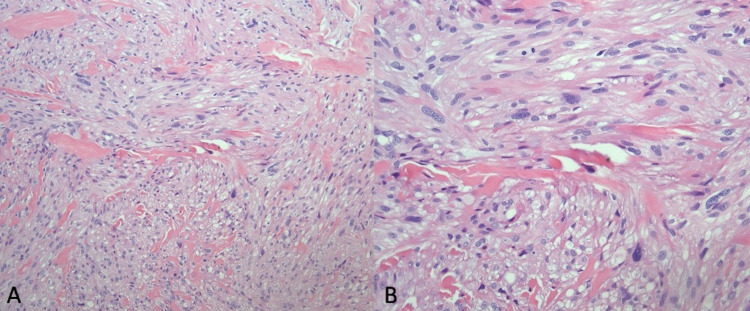
High power view at 20x (A) and 40x (B) showing highly atypical spindle cells with pleomorphic nuclei and mitotic activity.

**Figure 4:**
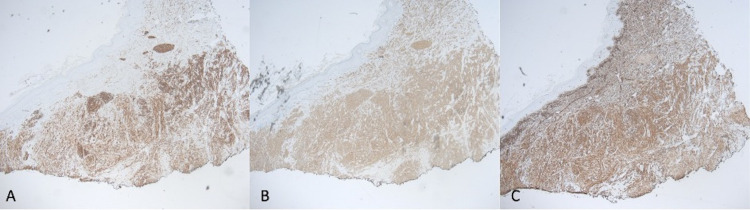
Immunostains show positive staining for desmin (A), smooth muscle actin (B), and vimentin (C) under low power view at 4x.

**Figure 5:**
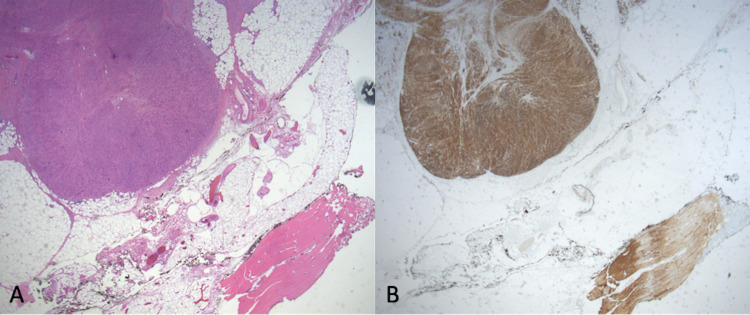
H&E stain (A) and immunostain of desmin (B) under low power view at 10x of the resection site.

Laboratory investigations are significant for a hemoglobin of 17.8 grams per decilitre and a white blood count of 6,000 per cubic millimeter of blood. The section shows highly atypical spindle cells with pleomorphic nuclei and mitotic activity on hematoxylin and eosin stains (Figures 2A, 2B). The biopsy section extends from the epidermis to the deep dermis (Figure 2C). The tumor cells are arranged in long and short fascicles (Figures 3A, 3B). Positive staining is present for smooth muscle actin, vimentin, and desmin immunostains (Figures 4A-4C, 5A, 5B). No staining is observed for cytokeratin AE1/3, p63, SOX10, S100, HMB45, CD68, CD10, or CD31. Based on the Fédération Nationale des Centres de Lutte Contre Le Cancer, histology supports a grade I leiomyosarcoma due to the presence of a well-differentiated tumor with a low mitotic rate (six mitoses per 10 high-power fields) and an absence of necrosis [6].

**Figure 6:**
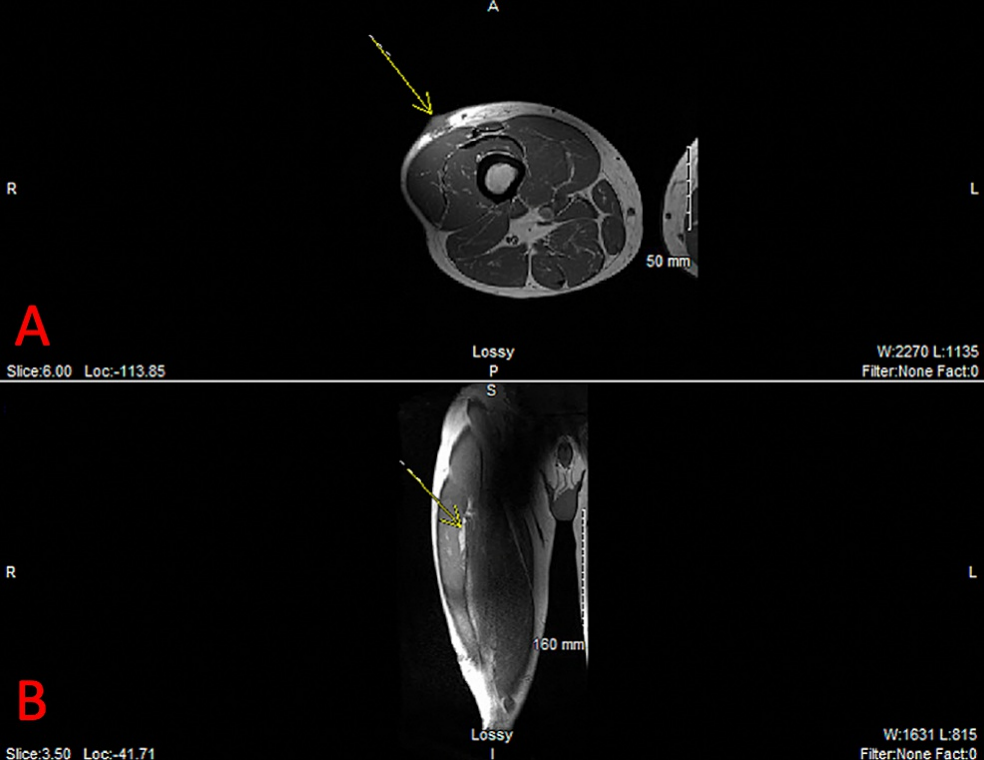
Enhancing lesion in the right anterolateral distal thigh with associated skin thickening and extension of the subcutaneous soft tissue in transverse dimension, marked by yellow arrow (A). Enhancement extends along the superficial fascial margin of the vastus lateralis muscle without definitive muscle invasion in the frontal plane, marked by yellow arrow (B).

Right femur MRI with contrast shows enhancing skin lesion in the anterolateral distal thigh extending into the subcutaneous soft tissue with skin thickening measuring 2.6 x 1.4 x 3.0 cm (Figures 6A, 6B). Nonspecific right inguinal lymph nodes were also noted on imaging. In order to assess the possibility of metastatic disease, CT imaging studies of the chest, abdomen, and pelvis were ordered. Chest CT scan with contrast is significant for a 5.6 mm non-calcified left upper lobe pulmonary nodule. Pulmonary function tests determine the functional expiratory volume (FEV1) of 47%.

The patient is treated with wide local excision of the right lower extremity under general anesthesia. An ulcerated tan-yellow-gray circumscribed mass with skin ellipse measuring 5 cm x 4.2 cm x 2 cm is obtained. Pathology confirms negative margins with 1.7 cm from the medial margin, 1.9 cm from the caudal margin, and 0.5 cm from the deep margins. No lymph nodes are identified.

At one-week follow up, the incision appears to be healing well with a small amount of sanguineous drainage. The wound is redressed in the office with an elastic bandage. The patient reports having ceased smoking since the procedure. At subsequent one-month follow-up, the incision appears dry with no erythema. Sutures are removed and dressing changed at an office visit. The patient is scheduled for follow-up over the next two years quarterly.

## Discussion

Primary cutaneous leiomyosarcoma is a rare soft tissue sarcoma that is believed to occur due to the malignant transformation of arrector pili muscles in the dermis. While cutaneous leiomyosarcoma is typically present at a lower stage with a five-year disease-specific survival of 98%, high-grade leiomyosarcoma can see a drop in survival to 50% after five years and requires more immediate treatment [7]. On presentation, patients typically complain of increasing pain associated with the sudden growth of the mass along with other findings such as ulceration, paresthesia, and bleeding. According to our knowledge, over the last 10 years, there are 11 other reported cases in the literature based on a PubMed search (Table 1). Of note, many of the cases report a previous history of local trauma, chronic non-healing ulcer, or prior leiomyosarcoma.

**Table 1:** A literature review of recent cutaneous leiomyosarcoma case reports. Abbreviations: LMS, leiomyosarcoma; WLE, wide-local excision; LFS, Li-Fraumeni syndrome.

Year	Sex	Onset Age	History	Site	Size	Grade	Treatment	Margins	Recurrence	Reference
2014	M	79	Local Trauma	Anterior Trunk	4 cm	I	WLE	2 cm	_______	[1]
2013	M	70	Chronic Ulcer	Lower Extremity	5 cm	III	WLE	5 cm	_______	[2]
2018	M	60	Chronic Ulcer	Lower Extremity	9 cm	____	WLE	_____	_______	[8]
2018	M	30	None	Anterior Trunk	5 cm	III	WLE	1 cm	72 months	[9]
2015	M	27	Previous LMS	Lower Extremity	7 cm	II	WLE	1 cm	24 months	[10]
2018	M	59	None	Upper Extremity	1 cm	____	WLE	_____	_______	[11]
2018	W	32	LFS	Upper Extremity	2 cm	I	WLE	_____	_______	[12]
2011	M	74	None	Posterior Trunk	3 cm	____	WLE	_____	_______	[13]
2018	F	54	Local Trauma	Face	5 cm	III	WLE	_____	12 months	[14]
2012	M	66	Local Trauma	Scapula	___	I	WLE	1 cm	_______	[15]
2021	F	93	Previous LMS	Upper Extremity	11 cm	III	Amputation	_____	36 months	[16]
2021	M	59	None	Lower Extremity	1 cm	I	WLE	1 cm	_______	

As the typical presentation of leiomyosarcoma is nonspecific clinically, definitive diagnosis depends on a histopathological skin biopsy. On H&E stain, the atypical spindle-shaped cells with cigar-shaped nuclei and eosinophilic cytoplasm are often arranged in poorly circumscribed fascicles. Typically, more than two mitoses per 10 high-power fields are identified. Immunohistochemistry of vimentin and smooth muscle actin is present in all cases, while desmin is present in over half of cases [3]. S-100 immunostaining has rarely been observed [17]. Kaddu et al. have described two primary histopathological growth patterns: nodular or diffuse [3]. Nodular is characterized by high cellularity, lots of mitotic figures, and the presence of necrosis whereas diffuse is characterized by low cellularity, few mitotic figures, and the absence of necrosis [3].

Cutaneous leiomyosarcoma is primarily treated with wide local excision, preferably with 1 cm margins in order to promote disease-free survival. In a review of 112 cases, Aneiros-Fernandez et al. found rates of recurrence to be 36.63% after an average follow-up time of 4.40 years [5]. Increasingly Mohs surgery for leiomyosarcoma has increased in popularity, due to closer margin control and lower recurrence rates. Murphy-Chutorian et al. report recurrence rates of 2.08% to 6.25% compared to 30% to 50% with wide local excision [18]. Mohs surgery has not been widely adopted due to few long-term prognostication differences, particularly for deeper lesions. Both radiotherapy and chemotherapy seldom are incorporated as adjuvant modalities. Due to inadequate studies, the role of adjuvant therapy has remained anecdotal, with the usage of either chemotherapy or radiation reserved primarily for distant metastases or recurrence [4]. Doxorubicin, ifosfamide, gemcitabine, or taxotere are effective in controlling progression, however, they are not effective at curing metastatic disease [19]. 

Due to high levels of recurrence of cutaneous leiomyosarcoma, regular follow-up is necessary. There is currently no clear standard for aftercare. However, Massi et al. recommend a follow-up quarterly for the first two years followed by a biannual visit for three years, and finally an annual visit for the next 20 years [20]. Radiography for possibly recurrence and metastases are included as part of a regular workup.

Limitations

Due to short follow-up, our study is limited in scope. Molecular studies were not performed as part of the workup for our case.

## Conclusions

An enlarging and tender mass should raise suspicion for cutaneous leiomyosarcoma with histopathological workup required to rule out the diagnosis. Although Mohs microsurgery or the addition of adjuvant therapy has become increasingly used, limited studies are available to merit its use over wide local excision alone. Follow-up, however, should remain close to prevent malignant disease progression.
